# High-Resolution Magic Angle Spinning (HRMAS) NMR Identifies Oxidative Stress and Impairment of Energy Metabolism by Zearalenone in Embryonic Stages of Zebrafish (*Danio rerio*), Olive Flounder (*Paralichthys olivaceus*) and Yellowtail Snapper (*Ocyurus chrysurus*)

**DOI:** 10.3390/toxins15060397

**Published:** 2023-06-15

**Authors:** Mark Annunziato, Narmin Bashirova, Muhamed N. H. Eeza, Ariel Lawson, Daniel Benetti, John D. Stieglitz, Jörg Matysik, A. Alia, John P. Berry

**Affiliations:** 1 Institute of Environment, Department of Chemistry and Biochemistry, Florida International University, Miami, FL 33181, USA; mannu002@fiu.edu (M.A.); alawson@fiu.edu (A.L.); 2 Institute for Analytical Chemistry, University of Leipzig, 04103 Leipzig, Germany; narmin.bashirova@medizin.uni-leipzig.de (N.B.); m.noureeza@gmail.com (M.N.H.E.); joerg.matysik@uni-leipzig.de (J.M.); 3 Institute for Medical Physics and Biophysics, University of Leipzig, 04107 Leipzig, Germany; a.alia@chem.leidenuniv.nl; 4 Department of Marine Biology and Ecology, Rosenstiel School of Marine, Atmospheric & Earth Science, University of Miami, Miami, FL 33149, USA; dbenetti@rsmas.miami.edu (D.B.); jstieglitz@earth.miami.edu (J.D.S.); 5 Leiden Institute of Chemistry, Leiden University, 2333 Leiden, The Netherlands

**Keywords:** nuclear magnetic resonance (NMR), metabolomics, fish, zebrafish, mycotoxins, zearalenone

## Abstract

Zearalenone (ZEA) is a mycotoxin, commonly found in agricultural products, linked to adverse health impacts in humans and livestock. However, less is known regarding effects on fish as both ecological receptors and economically relevant “receptors” through contamination of aquaculture feeds. In the present study, a metabolomics approach utilizing high-resolution magic angle spinning nuclear magnetic resonance (HRMAS NMR) was applied to intact embryos of zebrafish (*Danio rerio*), and two marine fish species, olive flounder (*Paralichthys olivaceus*) and yellowtail snapper (*Ocyurus chrysurus*), to investigate the biochemical pathways altered by ZEA exposure. Following the assessment of embryotoxicity, metabolic profiling of embryos exposed to sub-lethal concentrations showed significant overlap between the three species and, specifically, identified metabolites linked to hepatocytes, oxidative stress, membrane disruption, mitochondrial dysfunction, and impaired energy metabolism. These findings were further supported by analyses of tissue-specific production of reactive oxygen species (ROS) and lipidomics profiling and enabled an integrated model of ZEA toxicity in the early life stages of marine and freshwater fish species. The metabolic pathways and targets identified may, furthermore, serve as potential biomarkers for monitoring ZEA exposure and effects in fish in relation to ecotoxicology and aquaculture.

## 1. Introduction

**Figure 1:**
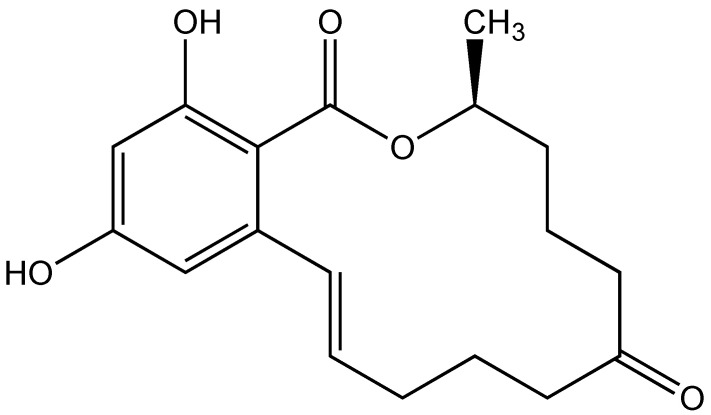
Structure of the mycotoxins, zearalenone (ZEA).

Mycotoxins produced by the genus *Fusarium* are found throughout the world, contaminating over 20% of the worldwide supply of agricultural commodities, particularly including grains such as maize, wheat, and barley [1]. One of the most commonly encountered mycotoxins in contaminated cereals is the xenoestrogen, zearalenone (ZEA; Figure 1). A recent, comprehensive global survey of mycotoxins in animal feeds, for example, reported detectable levels of ZEA in 20–58% of samples analyzed and median levels of 55 µg kg^−1^, but reaching, in some cases, 105 mg kg^−1^ [2]. As the main receptors of contaminated cereal products, mammals, including humans and livestock, have been the focus of the majority of studies concerning the adverse effects of ZEA exposure, and there remains to be significantly less known about the toxicological effects on aquatic organisms such as fish. 

With respect to the impact on fish, the introduction of ZEA into the aquatic environment is suspected to be mainly caused by run-off from farmland that contains *Fusarium*-contaminated crops and soil [3,4,5]. Due to the high stability of ZEA in the aquatic environment, concentrations as high as 220 ng L^−1^ have been measured in wastewater, rivers, and lakes [6]. In addition, fish have been increasingly found to be exposed to ZEA through commercial fish feeds used in aquaculture [7,8]. Current recommendations from governing bodies, such as the European Commission, state that the content of ZEA in livestock feeds should not exceed 2 mg kg^−1^, but no limits have been established for non-mammalian species such as fish [9]. Yet, levels of ZEA have been regularly detected in aquaculture feeds at levels more than 0.5 mg kg^−1^, and have been detected as high as 5.3 mg kg^−1^ in cereals destined for aquaculture feed production [7,10]. 

Showing a high affinity for estrogen receptors, ZEA toxicity has been historically associated with hormonal effects and the regulation of genes involved in cell survival and apoptosis in both in vitro and in vivo experiments [11]. The biological consequences of ZEA exposure have been studied using an array of fish species, as well as various in vitro systems [12,13,14]. Commonly reported effects have included altered reproductive function, such as intersex males, disrupted sperm production, and morphological deformities in early life stages [12,14]. Reproductive alterations resulting from ZEA exposure have been largely associated with the xenoestrogen properties of the compound and its high affinity for estrogen receptors (ER) in hormone-sensitive tissues [15,16]. Through these experiments, ZEA and some of its metabolites have been shown to bind to fish ER at more than 10-fold higher affinity than to human ER [17,18]. Mammalian cell lines, however, have shown similar cytotoxicity to ZEA, despite having differently expressed ER, suggesting that there are additional pathways responsible for the toxicity of ZEA. Moreover, cultured fish cells without detectable expression of ER, such as carp brain cell-line (CCB), have demonstrated high sensitivity to ZEA [13]. 

Alternative ZEA targets have, thus, been suggested through in vitro experiments utilizing various fish cell-lines and include a substantial increase in intracellular oxidative stress as a result of disrupted cell homeostasis [13]. Oxidative stress as an underlying mechanism of ZEA toxicity has, in particular, been reported by Muthulakshmi et al. [19], whereby zebrafish embryos exposed to ZEA showed significantly elevated levels of reactive oxygen species (ROS), lipid peroxidation and nitric oxide, and reduced levels of antioxidant biomarker responses including superoxide dismutase (SOD), glutathione (GSH) and glutathione-S-transferase (GST). Muthulakshmi et al. [19] were also the first to report a potential neurotoxic action of ZEA in fish, as acetylcholine esterase (AChE) activity was inhibited in exposed zebrafish embryos. Wozny et al. [20] examined the effects of ZEA exposure on the liver and ovary of rainbow trout through quantitative PCR and identified differentially regulated genes involved in DNA repair, cell cycle control, glycolysis, blood coagulation, and cytoskeleton structure. Although these previous studies have provided much insight into the toxicity of ZEA in fish, there remains a lack of *systems-level* understanding of ZEA toxicity that effectively identifies how these diverse effects are interrelated. 

Early life stages, including embryos and larvae of fish, have become widely established as both toxicological models and *ecological receptors*, as these vulnerable stages of development often occur in close proximity to surface waters and/or demersal zone, where many toxicants are concentrated [21]. Perhaps the most notable species used for toxicological assessment is the zebrafish (*Danio rerio*), a tropical freshwater cypriniform species native to shallow water systems such as ponds, canals, and rice paddies in southeastern Asia [22]. The popularity of zebrafish for toxicological assessment is due to several favorable traits such as their relatively small size (<1 mm), ability to spawn year-round, and nearly transparent embryo. At the same time, zebrafish may, in fact, serve as a relevant ecological receptor of ZEA, as run-off from agricultural fields, especially mycotoxin contamination of rice paddies (where zebrafish are frequently found), has been reported [23]. 

Although the zebrafish has proven to be an excellent *laboratory* model, laboratory-bred lines of zebrafish are not representative of the genetic, biochemical, and physiological diversity of “wild-type” fish. To better understand, therefore, the toxic effects of ZEA across the genetic diversity of ecologically relevant fish, particularly including species relevant to both aquaculture and fisheries, two marine species, namely olive flounder (*Paralichthys olivaceus*, Order: Pleuronectiformes) and yellowtail snapper (*Ocyurus chrysurus*, Order: Perciformes), were assessed alongside zebrafish in the present study. Both species may be relevant receptors of ZEA as aquaculturally relevant species (potentially impacted by contamination of feeds) and, in the latter case, a near-shore coastal species, potentially impacted by agricultural run-off. 

In the present study, we specifically employed a recently developed metabolomics approach based on the use of *high-resolution magic angle spinning* nuclear magnetic resonance (HRMAS NMR) to identify metabolic perturbations in embryonic stages of both laboratory lines of zebrafish and from spawning broodstocks of the two marine species in relation to ZEA exposure. Previously developed using the zebrafish embryo model and applied to a range of environmental toxicants [24,25,26,27,28,29], HRMAS NMR is capable of identifying and characterizing relevant metabolic pathways, including impacts of exposure to toxicants in intact embryonic and larval stages, enabling both integrated systems-level model of toxicity, and identification of relevant biomarkers of effect and exposure. The present study is the first to apply this technique to understanding the toxicity of ZEA in early life stages of fish, as relevant receptors and vertebrate models, and moreover, to extend both toxicity assessment and this powerful metabolomics technique to wild-type representatives of ecologically and economically relevant species. 

## 2. Results

### 2.1. Embryotoxicity of ZEA

**Figure 2:**
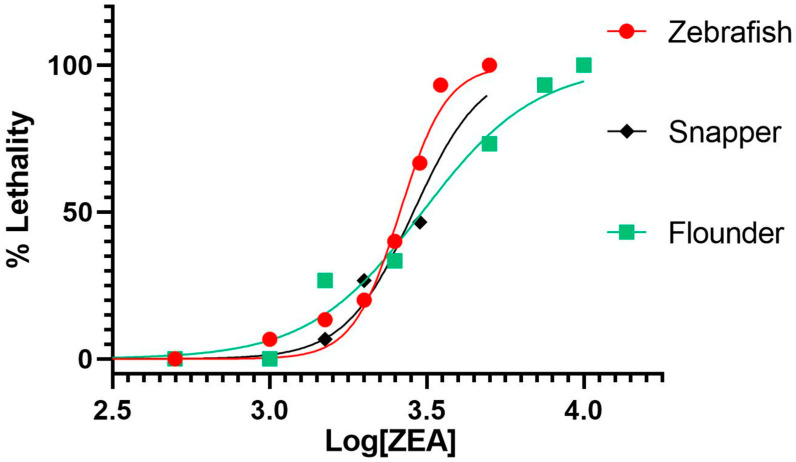
Dose-dependent toxicity of ZEA in embryos of zebrafish, snapper (i.e., yellowtail snapper), and flounder (i.e., olive flounder). Toxicity is measured as percent mortality (“% Lethality”) of embryos (N = 3 replicates of 5 embryos [n = 5]). Log concentration of ZEA is in parts-per-billion (ppb, i.e., µg L^−1^).

To assess acute toxicity and to establish suitable exposure concentrations, as well as other relevant parameters (e.g., developmental stage), for subsequent NMR-based metabolomics, embryotoxicity (and, specifically, lethality) of ZEA was evaluated in embryonic zebrafish, olive flounder and yellowtail snapper. Embryos were exposed for 24 h to a range of concentrations of ZEA immediately post-hatch, which corresponds to approximately 72 h post-fertilization (hpf) for zebrafish and flounder, and 24 hpf for snapper, in order to allow for the development of major organ systems (e.g., liver, CNS); subsequently, embryo survivorship was assessed as a measure of embryotoxicity. As shown in Figure 2, dose-dependent toxicity (based on percent mortality) of ZEA was observed for all three species, and ZEA was lethal to embryos of all species at concentrations above 1 ppm with no significant mortality (compared to solvent-only controls) at or below this nominal concentration. Notably, there was no significant difference between the calculated LC_50_ values between flounder (95% C.I. = 3.43–3.54 ppm), zebrafish (95% C.I. = 3.39–3.44 ppm), and snapper (95% C.I. = 3.42–3.59 ppm). Without a significant difference in response to ZEA exposure between these fish species, an exposure concentration of 1 ppm was selected, for all species, for subsequent HRMAS NMR experiments to avoid any non-specific effects on metabolite profiles caused by embryo mortality and morbidity while assuring, at the same time, an exposure concentration sufficient to elicit a measurable metabolic response. 

### 2.2. Altered Metabolic Profiles of Embryos Exposed to ZEA

**Figure 3:**
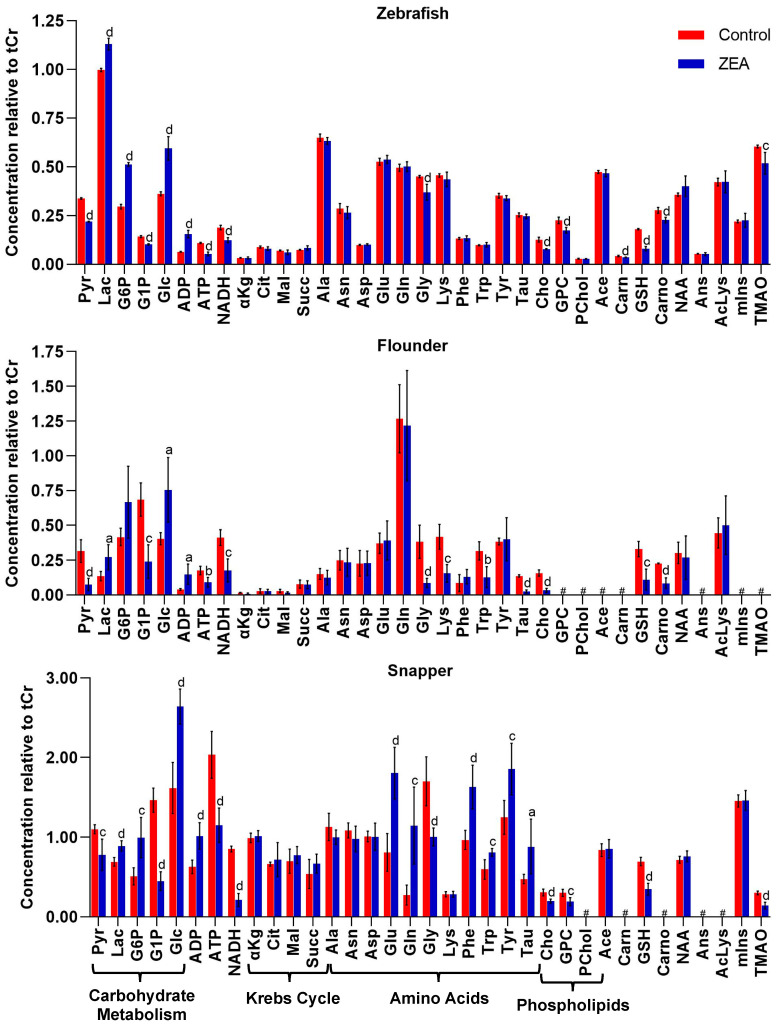
Measured levels of metabolites resolved by HRMAS NMR in zebrafish, flounder, and snapper. Metabolites include those associated with energy metabolism (e.g., carbohydrate metabolism, Krebs cycle, ATP/ADP, and NADH), amino acids, and phospholipids (associated with cellular membranes), among others, as described in the text. Shown are mean levels (±standard deviation), normalized relative to total creatine (creatine and phosphocreatine), for solvent-only “Control” embryos and zearalenone (“ZEA”) exposed embryos. Statistical significance of difference indicated with letters as follows: a, *p* < 0.05; b, *p* < 0.01; c, *p* < 0.005; d, *p* < 0.001. The label “#” indicates that the metabolite was not effectively resolved, and thus, quantified, from NMR spectra. Abbreviations: Pyruvate (Pyr), lactate (Lac), glucose-6-phosphate (G6P), glucose-1-phosphate (G1P), glucose (Glc), adenosine diphosphate (ADP), adenosine triphosphate (ATP), reduced nicotinamide adenine dinucleotide (NADH), a-ketoglutarate (αKG), citrate (Cit), malate (Mal), succinate (Succ), alanine (Ala), asparagine (Asn), aspartate (Asp), glutamate (Glu), glutamine (Gln), glycine (Gly), lysine (Lys), phenylalanine (Phe), tryptophan (Trp), tyrosine (Tyr), taurine (Tau), choline (Cho), glycerophosphorylcholine (GPC), O-phosphocholine (PChol), acetate (Ace), carnitine (Carn), reduced glutathione (GSH), carnosine (Carno), N-acetylaspartate (NAA), anserine (Ans), acetyllysine (AcLys), myo-inositol (mIns), and trimethylamine-N-oxide (TMAO).

**Figure 4:**
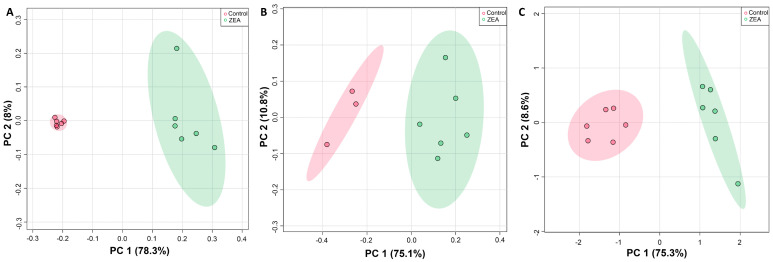
Principle components analysis (PCA) showing significant difference in metabolite concentrations (as measured by HRMAS NMR) of ZEA treated (green) versus solvent-only control (pink) embryos of zebrafish (**A**), flounder (**B**), and snapper (**C**). For all exposures, N = 6 except for solvent-only controls of flounder for which N = 3.

**Figure 5:**
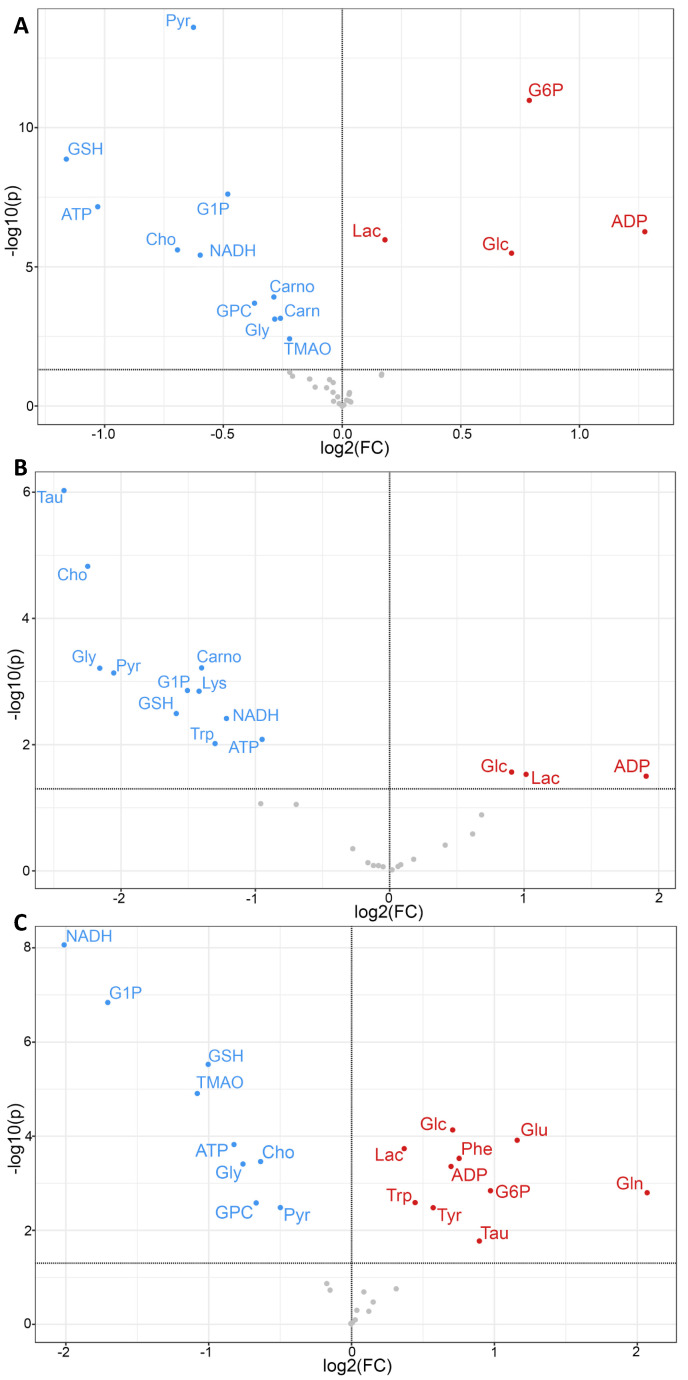
Volcano plots of metabolite changes in ZEA-exposed embryos of zebrafish (**A**), flounder (**B**), and snapper (**C**), compared to solvent-only control embryos, as measured by HRMAS NMR. Shown is log fold-change (FC) in metabolite, as either increase (red, >0), decrease (blue, <0) or no significant change (gray) relative to control, and level of significance (as log *p*-value) in the *y*-axis with minimal significance level (*p* < 0.05) shown as solid line. For abbreviations of metabolites, see Figure 3.

A minimum of 32 metabolites were resolved (Figures S1–S3) and quantified (Table S1) for each species by HRMAS NMR, with the largest number (39) measured in zebrafish embryos (Figure 3). Additionally, of these, 30 metabolites were effectively resolved and measured in all three species. Principal components analysis (Figure 4), and subsequent statistical analyses, revealed significant differences between treated and control, i.e., solvent-only, embryos, and specifically identified significant difference (Figure 5) for 23 of these metabolites of which 14 were consistently found to be altered, i.e., increased or decreased, for all species for which they were measured, including 10 which were both significantly and similarly altered for *all three species*. Whereas seven metabolites were found to be significantly, and similarly, changed for only one or two of the species measured, in only two cases, and specifically, the amino acids, tryptophan (Trp) and taurine (Tau), were opposing changes observed between species (increased in snapper, and decreased in flounder; Figure 3).

Of the 14 metabolites altered similarly in all species measured, significant increases were observed for several related to energy metabolism, including lactate (Lac), glucose (Glc), and adenosine diphosphate (ADP), whereas significant decreases were observed for metabolites associated, likewise, with energy metabolism including adenosine triphosphate (ATP), glucose-1-phosphate (G1P) and pyruvate (Pyr), as well as reduced forms of nicotinamide adenine dinucleotide (NADH). Other metabolites which were significantly, and consistently, altered by ZEA exposure include Choline (Cho), and two metabolites—namely, reduced glutathione (GSH) and its biosynthetic precursor, glycine (Gly)—associated with cellular defense, and specifically, oxidative stress and detoxification. In addition, for one or more species, significant increases were observed for glucose-6-phosphate (G6P), and amino acids including glutamate (Glu) and glutamine (Gln), and the aromatic tyrosine (Tyr), phenylalanine (Phe), whereas decreases were observed for lysine (Lys), carnitine (Carn), carnosine (Carno) and the liver-specific metabolite, trimethylamine N-oxide (TMAO).

### 2.3. LC-MS Lipidomics

**Figure 6:**
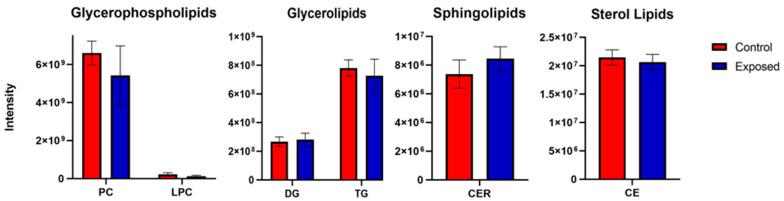
LC-MS lipidomics measurement of several lipid classes in ZEA-exposed (“Exposed”) versus solvent-only control (“Control”) embryos of zebrafish. Lipid classes evaluated include glycerophospholipids, and specifically, phosphatidyl choline (PC) and lysophosphatidyl cholines (LPC); glycerolipids, and specifically, triglycerides (TG) and diglycerides (DG); sphingolipids, and specifically, ceramides (CER); and sterol lipids, and specifically, cholesterol esters (CE).

To assess possible effect on lipid profiles, specifically in relation to membrane lipids (and given observed effects of ZEA on phosphatidyl choline components, i.e., Cho, GPC), a non-targeted analysis of several lipid classes was done. Although a decrease in phosphatidyl choline lipids was observed, the difference between ZEA-treated and control embryos was not significant (Figure 6). Non-significant differences were also observed for glycerolipids, sphingolipids, and sterols.

### 2.4. Visualization of ROS in ZEA-Exposed Zebrafish Embryos

**Figure 7:**
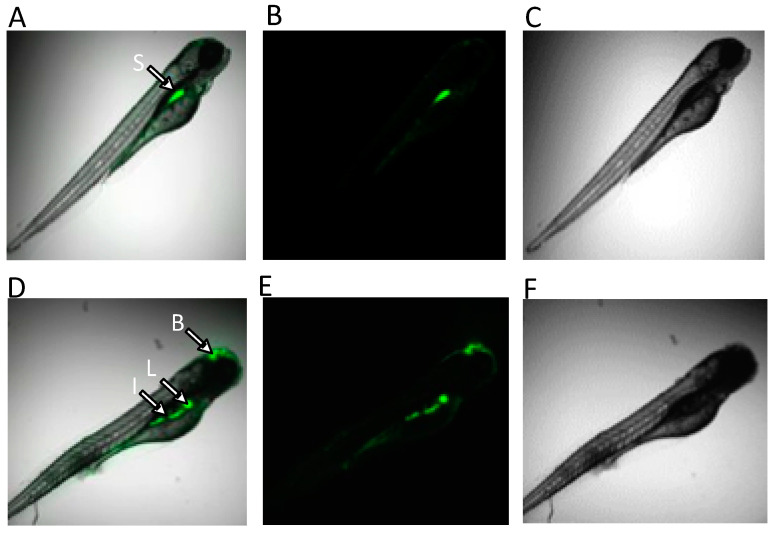
In vivo fluorescence detection of ROS in zebrafish embryos at 96 hpf, following 24 h exposure to ZEA (1 ppm), by confocal microscopy. As indicated, fluorescence (indicative of ROS) was detected in the GI tract, including stomach (S) or intestine (I) of both solvent-only control (top row) and ZEA-treated (bottom row) embryos, whereas fluorescence (indicative of ROS) was exclusively detected in the liver (L) and brain (B) region of ZEA-treated embryos. Shown are composite images of ZEA-exposed (**A**,**D**) of fluorescence (**B**,**E**) and light micrographs (**C**,**F**).

Given the suggested role of oxidative stress in the observed metabolic effects of ZEA (see below), reactive oxygen species (ROS) were visualized by fluorescent assay in the zebrafish embryo model as a means of localizing the primary organ systems affected. Whereas the production of ROS was observed in the gastrointestinal tract (i.e., stomach and intestine) in both solvent-only control and ZEA-treated embryos, elevated production of ROS in both the liver and brain region was additionally observed in ZEA-treated embryos (Figure 7).

## 3. Discussion

Extending previous studies of ZEA in the zebrafish embryo model [12,19,30,31], the present study investigated the toxicity of ZEA in embryonic stages of not only zebrafish but two additional marine species, namely olive flounder and yellowtail snapper. Additionally, it is, indeed, the first study to assess toxicity in these two ecologically and economically relevant species. Dose-dependent embryotoxicity was observed for all three species (Figure 2) with toxic concentrations (e.g., LC_50_ ≅ 3.5 ppm) comparable to those previously reported for the zebrafish embryo model [30,31]. Notably, snapper embryos develop more rapidly than zebrafish and flounder, with hatching and development of all major organ systems occurring within approximately 24 hpf, compared to 72 hpf, for the latter two species. Accordingly, both toxicity assessments and, subsequently, exposures for NMR metabolic profiling were conducted at 24 and 72 hpf, respectively. For subsequent NMR studies, embryos were specifically exposed at 1 ppm ZEA: this sub-lethal (<LC_50_) exposure concentration was selected to ensure a metabolic response but avoid any non-specific metabolic changes associated with the mortality and morbidity of embryos. While this exposure concentration is considerably higher than environmentally relevant concentrations of ZEA reported in waters that are typically less than parts-per-billion concentrations [6], ZEA in aquaculture feeds have been reported at concentrations greater than 1 ppm, i.e., mg kg^−1^ [7,10].

**Figure 8:**
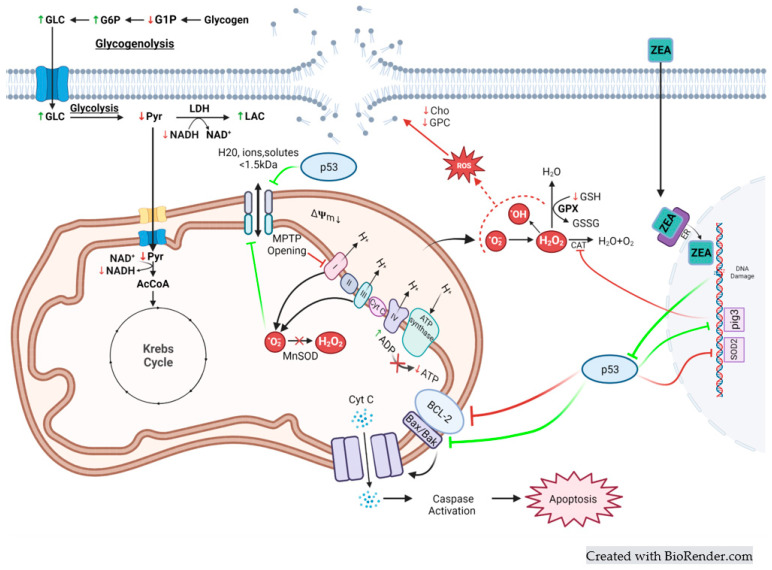
Integrated model of ZEA toxicity. Metabolites measured by HRMAS NMR, indicated with corresponding green upward arrows (↑) and red downward arrows (↓), indicating increase and decrease, respectively. It is known that ZEA interacts with estrogen receptors (ER) and, in turn, can translocate to the nucleus where genotoxic damage can induce expression of p53-dependent pathways, including (1) p52-inducible gene 3 (PIG3), which regulates catalase (CAT) activity, and superoxide dismutase 2 (SOD2) which encodes mitochondria manganese superoxide dismutase (MnSOD), which can consequently remove reactive oxygen species (ROS) via hydroperoxide, as well as (2) inactivation of B-cell lymphoma 2 (Bcl-2) that enables interaction of apoptotic factors Bak and Bax leading to the formation of openings in the mitochondria membrane, and subsequent leaks of cytochrome C, or alternatively, interaction with the mitochondrial permeability transition pore (MPTP). The latter can, in turn, impair the production of ATP by oxidative phosphorylation via disruption of the mitochondrial membrane potential (ΔΨm) and, subsequently, the electron transport chain (i.e., complexes I, II, III and IV, and cytochrome) and ATP synthase. At the same time, oxidative stress due to increased ROS can both impact cellular membranes, including mitochondrial membranes (and, consequently, MPTP) via lipid peroxidation with hydrolysis, and subsequent metabolism, leading to decreased levels of choline (Cho) and glycerophosphorocholine (GPC), as observed. The impact on mitochondria, in particular, can lead decoupling of the components of mitochondrial energy production, including, sequentially, the Krebs cycle and oxidative phosphorylation with increased consumption of NADH (decreased concentrations) despite decreased ATP production, as well as upstream impacts on carbohydrate metabolism including increased levels of glucose (Glc), as well as glucose-6-phosphate (G6P), due to either decreased flux through glycolysis to pyruvate (Pyr), and alternatively, into anaerobic glycolysis, and concomitant production of lactate (Lac) via lactate dehydrogenase (LDH), and/or alternatively, flux of Glc from glycogen (i.e., glycogenolysis) via glucose-1-phosphate (G1P) and G6P.

To further elucidate biochemical, molecular, and cellular pathways associated with ZEA toxicity, the present study utilized an NMR-based metabolomics approach, previously developed in the zebrafish embryo model, and adapted methodologies to embryonic stages of flounder and snapper, as ecologically and economically (i.e., aquaculture, fishery) relevant marine species. With respect to pathways of toxicity, ZEA is well known to bind, as a xenoestrogen, to ER and, consequently, modulate transcription of a number of estrogen-responsive genes [32], which include those associated with energy metabolism, such as glucose homeostasis [33] and mitochondrial function [34]. At the same time, structural mimicry of 17-β-estradiol (and other estrogens) by ZEA allows for binding to ER (and other steroid receptors), permitting translocation into the nucleus where ZEA can illicit DNA damage [35] through the formation of DNA adducts and DNA double-strand breaks [36,37]. Genotoxic damage induced by ZEA has been shown, in numerous studies [38,39,40,41,42,43,44,45,46,47,48], to subsequently lead to the activation of the TP53 gene and increase the expression of the transcription factor, p53, subsequently activating or repressing several genes that regulate cell cycle arrest, DNA repairs and response to oxidative stress, as well as mitochondrial function and dysfunction, and apoptosis. The involvement of p53-mediated molecular pathways, therefore, has likely relevance to a number of the cellular and biochemical pathways—and potential biomarkers [32,49]—associated with ZEA toxicity, including oxidative stress, and its corresponding consequences and responses, compromise of cellular membrane integrity and impairment of mitochondrial dysfunction, as well as consequent impacts on energy metabolism. Taken together with this prior knowledge regarding molecular and cellular targets of ZEA, alterations in the metabolic profiles of toxin-exposed embryos of the three species in the present study enabled the development of an integrated *systems-level* model (Figure 8).

### 3.1. Role of Oxidative Stress in ZEA Toxicity

A number of metabolites, and metabolic pathways, altered by ZEA, alongside direct observation of increased ROS production (Figure 7), suggest a role of oxidative stress in the toxicity of ZEA. Foremost, significantly decreased levels of GSH observed in ZEA-exposed embryos may reflect increased oxidative stress, as GSH is a key antioxidant within the cell [50]. Furthermore, decreased GSH in ZEA-exposed embryos was found concurrently with lowered Gly, a rate-limiting biosynthetic precursor that regenerates GSH levels through addition to glutamyl-cysteine [51,52]. Alternatively, lowered levels of GSH may be potentially attributed to Phase II detoxification of ZEA, as it has been demonstrated that glutathione-s-transferase conjugates GSH to an array of mycotoxins [53,54]. However, no study to date has shown GSH conjugation of ZEA or its metabolites, but rather glucuronidation and sulfonation [55]. Oxidative stress indices such as increased levels of lipid peroxidation and nitric oxide, alongside lowered GSH, in both fish cell cultures and embryos exposed to ZEA have, in fact, been observed in various studies, and various mechanisms have been suggested for ZEA-induced ROS, including unstable oxidative metabolites, DNA damage, and mitochondrial dysfunction [13,19].

High physiological levels of p53, as a possible consequence of ZEA-induced DNA damage, can modulate a number of genes that play a vital role in the oxidative response, particularly the p53-inducible gene 3 (PIG3). Previous reports have demonstrated that PIG3 regulates catalase activity through the direct binding of catalase, resulting in ROS generation in response to genotoxic damage [48]. On the other hand, p53 can also repress the expression of several genes, including the superoxide dismutase 2 (SOD2) gene that codes for the mitochondrial enzyme manganese superoxide dismutase (MnSOD). With the ability to catalyze the conversion of the superoxide radical (O_2_^•−^) into hydrogen peroxide (H_2_O_2_), MnSOD is one of the major cellular defenses against oxidative stress within the mitochondria, and the repression of this enzyme drastically decreases the ability to handle oxidative stress that can negatively impact the viability of cells [40] 

The role of oxidative stress in ZEA toxicity was further investigated in the present study through in vivo visualization of ROS production in zebrafish embryos. As shown in Figure 7, increased levels of ROS (compared to solvent-only controls) were found in both liver and brain regions of zebrafish embryos exposed to ZEA (whereas apparent constitutive ROS production was observed in the GI tract of both treated and control). Increased production of ROS in the liver is consistent with previous studies that have reported liver, kidney, and reproductive organs as targets of ZEA [56,57]. Notably, the liver in ZEA-exposed embryos was seemingly less developed, specifically observed as “budding” of the hepatic diverticulum, compared to the more fully developed liver in solvent-only control embryos, consistent (along with deformities, e.g., bending of body axis) with impairment of embryo development (Figure 7). Targeting of hepatocytes by ZEA is further suggested by decreased levels of TMAO (Figure 3), an established biomarker of hepatoxicity (produced exclusively in the liver), in both zebrafish and snapper. Similarly, decreased creatinine that has been linked to impaired liver function [58] was observed, although exclusively in snapper embryos, further suggesting the liver as a likely target of ZEA toxicity.

The observed increase in ROS in the *brain* region of zebrafish, on the other hand, aligns with a previous report [19] in which acetylcholinesterase (AChE) was inhibited in ZEA-exposed zebrafish, along with an observed increase in apoptotic cells in the brain region of exposed embryos, suggesting possible neuronal cytotoxicity of ZEA. Although AChE was not directly measured in the present study, Cho levels were decreased in all exposed groups, possibly resulting from the inhibition of AChE. Despite the indication of neuronal targeting by ZEA, however, no other metabolites associated with the CNS were found to be affected: most notably, N-acetylaspartate (NAA) was not altered by ZEA exposure in any of the three species (Figure 3). Localized almost exclusively to neurons, NAA is an established biomarker of neural damage and has been previously measured by HRMAS NMR in association with neurotoxicity in the zebrafish embryo model [25,28]. Although ZEA-exposed embryos of snapper had altered levels of Glu and Gln (which are part of the widespread postsynaptic excitation of neural cells), these changes may, indeed, relate perhaps more likely to their role in energy metabolism (as discussed below). 

### 3.2. Compromise of Cell Membrane Integrity by Oxidative Stress 

Potential consequences of oxidative stress and increased cellular ROS include the breakdown of cellular membrane integrity through oxidation of membrane proteins and lipids, particularly including *mitochondrial* membranes leading to impairment of function and the diffusion of the contents of the mitochondrial intermembrane space into the cytosol, such as cytochrome c, as previously reported for ZEA [59]. This can, in turn, promote mitochondria-mediated apoptosis [60]. Consistent with this, altered levels of metabolites associated with cell membrane structure and function were found to be altered in ZEA-exposed groups: specifically, both Cho and GPC levels were decreased significantly in the present study and are vital, as components of phosphatidyl choline (PC), to the structural integrity of cellular membranes, as well as having important functions in lipid transport, metabolism, and signaling processes [61]. Interrelated through the CDP-choline pathway [62,63], it is proposed that concurrent lowering of GPC and Cho may indicate disruption to the cell membrane and subsequent hydrolysis of phospholipids—as has been observed, likewise, in previous studies of ZEA in mammals [64,65]—followed by subsequent metabolism. As an essential metabolite (not synthesized to any significant extent in vertebrates), *decreased* levels of Cho and GPC are most likely due to either catabolism, or “recycling” as either acetylcholine or phospholipids (via CDP-choline, or Kennedy Pathway), or other (e.g., microbial) pathways. The exact metabolic fate(s) of Cho and GPC is, however, not clear from the current data, and remains to be clarified. 

To further investigate the possible effect of ZEA on lipids (as components of cell membranes), LC-MS lipidomics investigated several classes of membrane-associated lipids in zebrafish embryos (Figure 6). Although PC lipids were lower in ZEA-exposed embryos, the difference was not significant. Notably, o-phosphocholine as a biosynthetic precursor was unchanged in ZEA-exposed zebrafish (not measured in the other two species), suggesting no role of reduced biosynthesis of PC. Consistent with damage to the cellular lipid bilayer is significantly decreased levels of carnosine for both zebrafish and flounder (whereas it was not effectively resolved, nor thus, measured for snapper). This dipeptide is known to directly scavenge unsaturated aldehydes created by peroxidation of fatty acids from cell membranes through the formation of covalent adducts, as well as form charge-transfer complex with superoxide radicals during times of heightened oxidative stress [66,67]. Although lipid peroxidation was not directly measured, the increased ROS production, and concurrent changes in levels of metabolites associated with membrane phospholipids (i.e., Cho, GPC) and those which directly scavenge products of peroxidation of membranes (i.e., carnosine), suggests that disruption of membranes, as a consequence of oxidative stress, is likely to occur in ZEA-exposed embryos. 

### 3.3. Perturbation of Energy Metabolism by ZEA

Prominently, numerous features of the metabolic profiles observed in the present study point to the perturbation of energy metabolism. Most conspicuously, perhaps, significantly decreased ATP, alongside concomitant increases in ADP, in ZEA-exposed groups was observed in all three species (Figure 5). This imbalance in ATP/ADP ratio is most likely explained by impairment of mitochondrial function, particularly including disruption of the permeability of the mitochondrial membrane, perhaps as a result of oxidative stress (as discussed above), and specifically, hydrogen ion permeability, i.e., proton concentration gradient, and the electron transport chain, associated with the production of ATP by oxidative phosphorylation [68]. At the same time, a number of metabolites associated with energy production via mitochondria are likewise altered by ZEA exposure (Figure 3). Alongside decreased ATP, for example, NADH was significantly decreased in all three species: as the primary product of the Krebs cycle, NADH serves, in turn, as the primary electron donor for the electron transport chain and simultaneously, source of protons (for chemiosmosis), driving phosphorylation of ADP to ATP. As it has been shown that the “energy state” is largely determined by levels of ADP [69], it is likely perhaps that increased [ADP] drives oxidative phosphorylation and, consequently, consumption of NADH (without consequent production of ATP). This is further supported by the lack of significant change in any metabolites associated with the Krebs cycle, as measured in the present study (e.g., malate, citrate, σ-ketoglutarate, succinate; Figure 3), suggesting that the Krebs cycle itself is not affected. That said, a reduction in NADH may alternatively or additionally reflect consumption associated with other compensatory energy-producing pathways (e.g., glycolysis/gluconeogenesis; as discussed below).

Aligned with the increased energy demand that would result from a disruption to the mitochondria, and reduced production of ATP by oxidative phosphorylation, a simultaneous increase in Glc, as well as G6P (in zebrafish and snapper), and a decrease in G1P hint at alterations to glucose metabolism in relation to energy homeostasis. While Glc is generally associated with its vital role in glycolysis (to Pyr), G1P is almost exclusively associated with the metabolism of glycogen during glycogenolysis (and glycogenesis). The concurrent alterations of these two metabolites would be consistent with the metabolic flow from glycogenolysis (via G1P) to the production of Glc to meet energy requirements. On the other hand, G6P is associated (as intermediate) with both glycogenolysis, and glycolytic conversion of Glc to Pyr. Taken together, these findings are consistent with previous studies in mammals that revealed increased glucose utilization, and initiation of glycogenolysis, as a major metabolic effect of mycotoxins, including ZEA [64,70].

Alongside observed alterations in glucose metabolism, the downstream glycolytic product, Pyr, was found to be decreased in ZEA-exposed fish, whereas Lac notably increased significantly. Decreased Pyr, alongside both decreased NADH and increased Glc (and G6P), might suggest inhibition of glycolysis. Alternatively, decreased Pyr (i.e., increased consumption) would be similarly expected to occur, particularly in light of reduced ATP, as the metabolic flow into the Krebs cycle could potentially compensate for increased energy (and, specifically, ATP) production. However, if mitochondrial dysfunction occurs, as suggested, and energy demand exceeds the rate at which oxidative phosphorylation can provide sufficient ATP, Pyr may be instead converted to Lac by way of the anaerobic respiration pathway [71], and the NAD+ and Lac generated by this metabolic pathway utilized to supply, respectively, additional glycolysis and/or gluconeogenesis (in the liver via the Cori cycle). Coinciding, in fact, with the decrease in Pyr, and increase in Lac, was a significant decrease in NADH which may (alongside disruption of mitochondrial function) result from oxidation of NADH to NAD+ during the conversion by the enzyme lactate dehydrogenase (LDH). Taken together, it is proposed that observed changes in these metabolites suggest both decreased glycolysis and a shift to alternative energy-producing pathways (e.g., anaerobic glycolysis). 

Paralleling these alternative ATP-producing pathways, a significant change in carnitine (increase in zebrafish) and several amino acids (variable between species) were observed (Figure 5) and may likewise suggest compensatory metabolic pathways to offset decreased production of ATP via oxidative phosphorylation. In the former case, carnitine is almost exclusively associated with the transport of fatty acids to the mitochondria for β-oxidation (which produces both NADH and FADH_2_). When Glc is in excess, as observed here, synthesis of fatty acid in the liver is upregulated, and in turn, catabolic breakdown is inhibited, leading to the presumptive build-up of acylcarnitine and a consequent decrease in free carnitine as observed. With respect to amino acid catabolism, the observed decrease in Lys (specifically in flounder) is, perhaps, particularly telling: catabolism of Lys occurs in mitochondria through the saccharopine pathway, generating acetyl CoA for the Krebs cycle [72,73]. Additionally, the presumptive consumption of Lys to accommodate for lowered glycolytic Pyr would similarly suggest a metabolic shift to compensatory energetic pathways. That said, there appear to be considerable taxa-specific effects on amino acid metabolism and, thus, the possibly variable role of other compensatory pathways in the three species (as discussed below). 

The molecular pathways associated with changes in energy metabolism may include either those driven by ER (and ZEA as a xenoestrogen) or, alternatively, pathways induced via genotoxic damage and subsequent cellular response (e.g., apoptosis). It is known that estrogens, and in turn, ER, are intrinsically involved in the regulation of energy homeostasis, including mitochondrial activity [34], and both lipid and carbohydrate metabolism, including, in the latter case, glucose metabolism and subsequent glycolysis [33]. Alternatively, or additionally, impacts on energy metabolism may result secondarily from disruption of cellular function, including oxidative stress and associated mitochondrial impairment. The lowering of Pyr in exposed fish may, for example, result from caspase activity (resulting from mitochondrial dysfunction), as caspases are known to impair glycolysis via the glycolysis-limiting enzymes phosphofructokinase and pyruvate kinase [74]. Similarly, lower Pyr may be due to activated p53 that has been, likewise, shown to inhibit glycolysis via inhibiting HIF1α and inducing TP53-induced glycolysis and apoptosis regulator (TIGAR) [75]. Regardless of the mechanism, the majority of changes in metabolic profiles observed in the present study, as well as in similar studies [76,77], point to mitochondria as a central component of ZEA toxicity. 

### 3.4. Role of Mitochondrial Dysfunction in ZEA Toxicity

Numerous metabolites altered by ZEA (as measured by HRMAS NMR; Figure 3) suggest a central role of mitochondrial dysfunction in the proposed model of ZEA toxicity (Figure 8). Included among these are aforementioned metabolites associated with energy metabolism and homeostasis, for which mitochondria are of key importance, and metabolites associated with oxidative stress, e.g., GSH, alongside direct observation of increased ROS (Figure 7), and consequent disruption of cellular membranes including, perhaps, the mitochondrial membranes.

With respect to the latter, excessive ROS production within cells, or decreased ability to remove oxidative products, can result in *mitochondrial permeability transition pore* opening, as ROS can oxidize thiol groups of the MPTP complex [38]. The resultant depolarization of membrane potential from MPTP opening has, in fact, been noted previously in ZEA exposure studies [43,47], along with elevated levels of ROS. However, there remains debate if this change in membrane potential occurs from mitochondrial ROS accumulation or upstream of oxidative stress within mitochondria. Increased levels of superoxide within the cell may also trigger permeabilization of the outer mitochondrial membrane through a voltage-dependent anion channel (VDAC) dependent permeabilization process leading to free passage through the mitochondria of molecules of <1.5 kDa, including protons [42]. In addition, ROS alone is not the only suspect for mitochondrial disruption as ZEA may *directly* target the outer membrane VDAC [43], and p53 can interact with B-cell lymphoma 2 (Bcl-2 family) members, resulting in mitochondrial outer membrane permeabilization and the initiation of mitochondria-induced apoptosis [41]. The inactivation of Bcl-2, which binds the apoptotic factors Bak and Bax by p53, allows for the oligomerization of Bak/Bax and the formation of openings in the mitochondrial membrane, leading to the release of cytochrome c and the subsequent initiation of the apoptosis cascade [39,42]. Mitochondrial permeability induced by p53 can also come as a result of physical interaction with the MPTP regulator, cyclophilin D (CypD), leading to pore opening [46]. 

### 3.5. Interspecies Differences 

Species of embryos assessed in the present study span three orders of teleost fish, including Cyprinoformes (i.e., zebrafish), Perciformes (i.e., snapper), and Pleuronectiformes (i.e., flounder). However, alterations of metabolic profiles by ZEA were largely consistent between the species, with the relative change or lack thereof shared for 21 of 30 metabolites measured across all three species; this includes cases for which metabolites were only measurable in one or two (of the three) species, consistent changes were observed in 30 of the 39 cases (Figure 3). In seven cases, significant changes in metabolites were found in only one or two of the three species, and only two metabolites were *differentially* altered between species. It is proposed that observed differences may, in particular, reflect underlying interspecific differences in the compensatory processes associated with energetic metabolic challenge imposed by ZEA via impairment of mitochondrial function.

Most conspicuously, differential effects were observed in relation to several amino acids, including Glu and Gln, and the aromatic amino acids (AAA), i.e., Phe, Tyr, and Trp, as well as the similarly *essential* amino acid, Lys, and the non-proteinogenic Tau. Indeed, several studies have shown that amino acid metabolism, and in particular, catabolism in relation to energetic demands, is uniquely important in fish, including early life stages [78,79,80,81]. Catabolism of amino acids by teleost is substantially higher than in mammals, with Glu and Gln, in particular, serving as major fuels for energetic homeostasis [78]. At the same time, it has been suggested that AAA metabolism is particularly important during early development as precursors to a number of thyroid hormones that regulate embryogenesis and that the requirement of these amino acids for development is balanced with catabolism for energy metabolism [80,82]. Of the species assessed, only embryos of snapper showed increased levels of both Glu, Gln, and all three AAA. As Phe and Trp are essential amino acids for fish (and Tyr is biosynthesized from Phe), increased levels of AAA, in particular, point to decreased catabolism. The catabolism of AAA has been found to occur primarily in the liver, and thus, alterations (i.e., increase) in levels of AAA in the present study may be due to hepatotoxic effects caused by ZEA exposure as noted in previous studies [31,83]. The elevated levels of amino acids are further notable as a concomitant decrease in creatinine was observed for snapper: alongside creatinine as a recognized indicator of renal function, it has been shown that high AAA are linked—as precursors to uremic toxins—to kidney dysfunction [84]. 

In contrast, a decrease in Lys, as the second most important essential amino acid, and Trp in flounder suggests *increased* catabolism. Interestingly, flounder was the only species for which a significant change in G6P (relative to Glc) was not observed: recent studies have indicated that G6P-associated pathways, including glucose-6-phosphatase and glucose-6-phosphate isomerase, respectively, are regulated by amino acids [85], or their metabolites including saccharopine from Lys catabolism [86], suggesting interplay between amino acid and carbohydrate metabolic pathways. Additionally, it has been suggested that G6P is, more generally, a “central hub” for both energy metabolism including amino acids [87]. 

Notably, levels of the non-proteinogenic Tau were decreased in flounder but increased in snapper: whereas there is no known catabolic pathway for Tau, concentrations of this metabolite in fish are controlled by regulated biosynthesis from cysteine in hepatocytes [88]. Previous studies have, indeed, shown that Tau is biosynthesized de novo in zebrafish embryos and has an essential role in a number of biochemical, physiological, and developmental processes [89]. Additionally, the regulation of Tau biosynthesis with respect to rate-limiting steps, namely cysteine dioxygenase and cysteine sulfonate decarboxylase, has been studied in olive flounder and found to be relatively less sensitive to cysteine concentrations with lower expression and catalytic efficiency of the latter biosynthetic step [88]. It is suggested that decreased levels (in flounder) may, therefore, reflect the diversion of cysteine to energetic pathways, and in fact, the alternative fate of cysteine is catabolism to pyruvate. Alternatively, decreases may indicate reduced biosynthesis of the amino acid from its precursors, serine and methionine. In contrast, increased levels of Tau in snapper presumably reflect an increased biosynthesis. This is compelling in light of the well-documented role of Tau in reducing oxidative stress in mitochondria [90], as suggested to be a key component of ZEA toxicity. Coincidentally, in fact, an increase in Glu and Gln was specifically observed in ZEA-exposed snapper. Glutamate, well-known for being the most abundant excitatory neurotransmitter, is cycled between neurons and astrocytes via the Glu/Gln shuttle and, once released into synapses, subsequently binds and activates glutamate receptors that can initiate the postsynaptic influx of Ca^2+^ [91]. Among the antioxidant roles of Tau is the ability to regulate intracellular calcium homeostasis and protect against glutamate-induced excitotoxicity by reducing the influx of Ca^2+^ into neurons through synaptic neurotransmitter receptors [92]. It is, thus, tempting to speculate—given the observed increase in ROS in brain regions of zebrafish (Figure 7)—that increased Tau may serve a protective role in possible neural targeting of ZEA.

## 4. Conclusions

NMR-based metabolic profiling of embryonic stages of three fish species exposed to ZEA specifically identified alterations of metabolites associated with oxidative stress, energy metabolism, cellular membrane integrity, and disruption to mitochondrial function. These observed metabolic perturbations are consistent with recognized estrogenic effects of ZEA, as well as the previously reported role of p53 pathways, but also provided additional insight into less understood effects, including possible neurotoxicity. Additionally, taken together, enabled an integrated systems-level model providing unprecedented insight into the toxicity of ZEA (Figure 8). 

The present study is the first to compare ZEA toxicity, and associated metabolic responses, between three taxonomically distinct teleost fish, including cypriniform, perciform, and pleuronectiform species, to understand the role of phylogenetic diversity in ZEA toxicity. Additionally, moreover, the first to assess the effects of this mycotoxin—as a documented contaminant of coastal waters and aquaculture feeds—in relation to ecologically and/or economically relevant species relative to the established laboratory model of the zebrafish. Notable consistencies were observed in the metabolic profiles of the three species, but at the same time, several potentially noteworthy differences were observed. Future studies are, thus, warranted to further characterize and understand interspecific differences as they relate to the response to this widespread biotoxin and, simultaneously, identify both generalizable and taxa-specific biomarkers of toxicity (i.e., exposure and effect) as tools with potential utility for improved environmental monitoring, and assessment of exposure—and adverse effects—in marine and freshwater fish in the environment, and in aquaculture. 

## 5. Materials and Methods

### 5.1. Chemicals and Other Materials 

Zearalenone was purchased from Cayman Chemical (Ann Arbor, MI, USA). All other chemicals (i.e., deuterated phosphate buffer and reference standard for NMR) were purchased from Sigma-Aldrich (St. Louis, MO, USA) unless otherwise specified. 

### 5.2. Fish Rearing and Breeding

For assessment of toxicity and HRMAS NMR studies, fertilized eggs (i.e., embryos) of zebrafish (OBI/WIK line) were obtained from the Helmholtz Centre for Environmental Research UFZ (Leipzig, Germany). Rearing and breeding, and all subsequent experimental procedures (described below), for zebrafish, were performed according to previously described methods [25] and in accordance with the German animal protection standards approved by the Government of Saxony, Landesdirektion Leipzig, Germany (Aktenzeichen 75-9185.64), and guidelines of the European Union, Directive 2010/63/EU, which expressly permits the use of zebrafish embryos (up to 120 h hpf). 

For flounder and snapper, fertilized eggs were obtained from spawning broodstock at UMEH, following standardized UMEH protocols for marine fish species. Briefly, floating eggs were passively collected from broodstock maturation tanks (15–30 m^3^) in attached 400-L egg collector tanks following spawning events, as described for other marine fish species [93,94]. Subsequently, after collection in the egg collector tank, eggs were placed in a 5-L beaker filled with filtered and UV-sterilized seawater for 10 min to separate floating, viable eggs from sinking (and potentially non-viable) eggs. Floating, viable eggs were collected and kept in filtered seawater, in an incubator, at a density of 300 eggs/L; the incubator was equipped with a central standpipe fitted with a 300-μm mesh and supplied with pure oxygen and gentle ventilation (through an air ring placed at the bottom of the standpipe) with temperature maintained at 17 °C (flounder) and 26 °C (snapper), and dissolved oxygen maintained between 6.5 and 8.5 mg/L. Rearing and breeding of flounder and snapper, as well as subsequent experimental procedures (see below), were performed according to protocols approved by the University of Miami’s Institutional Animal Care and Use Committee (UM IACUC Protocol #20-138) and executed by trained personnel.

### 5.3. Assessment of Embryotoxicity of ZEA 

To determine appropriate, and specifically sublethal, exposure concentrations for HRMAS NMR experiments, and assess the toxicity of ZEA, in general, embryos of each of the three species (i.e., zebrafish, olive flounder, and yellowtail snapper) were exposed to a range of concentrations over a 24 h period, and assessed for lethal effects. For all three species, exposure times (i.e., 24 hpf or 72 hpf) were selected to coincide with hatching (loss of chorion) and the development of major organ systems.

For zebrafish, an exposure concentration range (0.5 to 5 ppm ZEA) was established in preliminary studies, and embryotoxicity was subsequently assessed, as per previous studies [28], in triplicate (N = 3) in polypropylene 24-well plates (Evergreen Scientific, Los Angeles, CA, USA) with each well containing 5 embryos (n = 5) in 1-mL of E3 medium [95]. Embryos were exposed at 72 hpf to allow the development of key systems (e.g., liver, CNS) and to avoid possible complications with chemical uptake (through chorion). After 24 h of exposure time (96 hpf), embryo lethality was assessed based on the cessation of movement and absence/presence of heartbeat using a dissecting light microscope as per previously established and validated protocols [28,96]. Methods to evaluate embryotoxicity in flounder and snapper were adapted, in turn, from these previously developed methods. 

To evaluate ZEA embryotoxicity in flounder, exposures were, similar to zebrafish, performed at 72 hpf (for a 24 h period) to likewise allow for the hatching and development of organ systems. Although hatching times are similar to zebrafish, several key modifications to the methods used for zebrafish were required. Prior to exposure, viable flounder embryos were selected from collected spawn: this was accomplished by “screening” of floating (and, thus, viable) eggs and subsequent selection by microscopic observation. At 48 hpf, viable embryos (in chorion) were placed in 35 mm Petri dishes with 5 mL of bio-filtered seawater (i.e., 10-μm filtered hatchery system water) into which ZEA was subsequently diluted at 72 hpf. The use of a larger volume of water for exposures, in comparison to zebrafish, was performed to circumvent the adverse impacts of high embryo density in test plates, as observed in preliminary studies. An exposure concentration range of 0.5 to 100 ppm ZEA was utilized based on preliminary observations, and exposures to ZEA (alongside solvent vehicle-only, i.e., 0.0001% ethanol, controls) were performed in triplicate (N = 3) with five embryos per dish (n = 5). Mortality was assessed using the same criteria (i.e., observable movement and heartbeat) utilized in zebrafish assays mentioned above. 

In contrast to zebrafish and flounder, snapper embryos develop and hatch much more rapidly (<48 hpf), and thus, exposures for embryotoxicity assessment were initiated at 24 hpf (continuing for a subsequent 24 h period) to allow similar developmental stages to be observed. Snapper embryos (24 hpf) were placed in 35 mm Petri dishes with 5 mL of bio-filtered seawater (i.e., 10-μm filtered hatchery system water) with ZEA over a concentration range 0.5 to 5 ppm, similar to that used for zebrafish. Each test plate included 5 embryos (n = 5), and each concentration was evaluated in triplicate (N = 3), and at 48 hpf, mortality was assessed based, likewise, on the absence of heartbeat and cessation of movement as determined using a light microscope. 

For each species, median lethal concentrations (LC50) were calculated using Probit analysis in SPSS (version 26.0; IBM Corporation, Armonk, NY, USA) and statistically compared based on the overlap of calculated 95% confidence intervals. All data were visualized using GraphPad Prism software v.9.2 (GraphPad Software, Inc., Boston, MA, USA). Toxicity assays were conducted at FIU under protocols approved by the FIU Animal Care and Use Committee (IACUC-19-085-AM01).

### 5.4. Exposure of Embryos and Sample Preparation for HRMAS NMR 

Exposures of embryos to ZEA for HRMAS NMR analysis were conducted based on previously established protocols [28] for zebrafish, whereby approximately 120 embryos in replicates at 72-hpf for zebrafish (N = 6) and flounder (N = 3), or 24-hpf for snapper (N = 6), were exposed for 24 h in 25-mL of either ISO medium (for zebrafish) or bio-filtered seawater (for flounder and snapper) in 100 mm polystyrene Petri dishes. Exposure times of 24 hpf and 72 hpf for snapper, and flounder and zebrafish, respectively, were selected to synchronize developmental stages between the more rapidly developing, i.e., snapper, and less rapidly developing, i.e., flounder and zebrafish, species (see Section 5.3. Assessment of embryotoxicity of ZEA). At the end of the 24 h exposure period, embryos were washed with MilliQ water to remove excess ZEA and media. Collected embryos were snap frozen (to −80 °C) until analysis. In the case of flounder and snapper embryos, exposures were performed at FIU (Miami, FL, USA), and frozen embryos were subsequently transported (on dry ice) to the laboratory at the University of Leipzig (Leipzig, Germany) for NMR analysis; for zebrafish, both exposure and NMR analyses were done at the University of Leipzig. Prior to NMR analysis, 100 embryos were transferred to a 4 mm zirconium oxide rotor (Bruker BioSpin AG, Switzerland) to which 10 μL of deuterated phosphate buffer (100 mM, pH 7.0) containing 0.1% (*w*/*v*) 3-trimethylsilyl-2,2,3,3-tetradeuteropropionic acid (TSP), as a chemical shift reference, was added. 

### 5.5. HRMAS NMR and Data Analysis 

NMR experiments in the present study were conducted using a Bruker DMX 600-MHz NMR magnet with a proton resonance frequency of 600 MHz, and equipped with a 4 mm HRMAS dual ^1^H/^13^C inverse probe with a magic angle gradient and spinning rate of 6 kHz, based on parameters previously optimized for zebrafish embryos [24,25,26,27,28,29]. Measurements were carried out at a temperature of 277 K using a Bruker BVT3000 control unit, while the acquisition and processing of data were conducted in Bruker TOPSPIN 4.0.6 software (Bruker Biospin GmbH, Ettlingen, Germany). Identification and quantification of metabolites were performed through Chenomx NMR Suite 8.2 (Chenomx Inc., Edmonton, AB, Canada) utilizing the Human Metabolome Database (HMDB) and the 600-MHz library (from Chenomx) which uses the concentration of a known reference signal (in our case TSP) to determine the concentration of individual compounds. The concentrations of metabolites were subsequently calculated based on a ratio relative to tCr (as previously described [26]. Statistical analysis of NMR quantification was done using Metaboanalyst 5.0 (https://www.metaboanalyst.ca/, accessed on 19 October 2022). Differences in individual metabolites were evaluated using a *t*-test with a *p*-value < 0.05 considered significant. Two-dimensional principal component analysis (2D PCA) scores plots with visualized 95% confidence regions were constructed using Metaboanalyst 5.0 software. 

### 5.6. LC-MS Lipidomics of Zebrafish Embryos

Zebrafish exposures for lipid analyses used previously established protocols, as described above for HRMAS NMR analyses, in which approximately 100 embryos in replicates (N = 3) were exposed to ZEA for a 24 h period. For the extraction of lipids, 100 embryos were homogenized in 1 mL of methanol:water (1:1, *v*/*v*) mixture with 10 µL of labeled internal standard (EquiSplash Lipidomix, Avanti Polar Lipids, Alabaster, AL, USA). Subsequently, 1 mL of chloroform was added to the sample and then sonicated for 15 min. The sample was then centrifuged at 4500 rpm for 20 min, and the chloroform layer was removed and dried under nitrogen gas. The sample was reconstituted with 50/50 Acetonitrile/Water before analysis. 

Chromatographic separation was achieved using an LC-20 CE ultrafast liquid chromatograph (Shimadzu, Japan) with an Accucore C30 column (Thermo Fisher Scientific, Sunnyvale, CA, USA). The mobile phase consisted of solvent “A” (30% ACN; 40% water; 30% IPA) and solvent “B” (10% ACN; 5% water; 85% IPA) with an elution gradient as follows: Sample injection at 0% B and hold for 1 min; from 1 to 5.6 min increase to 55% B and hold until 6.4 min; increase to 65% B at 6.4 min and hold until 24 min; increase to 88% B at 24 min until 40.8 min; increase to 95% B until 48.1 min; after 48.1 min, decrease to 35% B and hold until 56 min; decrease to 0% B at 56 min and hold until 60 min. The mobile phase flow rate was set at 200 μL/min with an injection volume of 5 µL. 

For MS/MS experiments, a TIMS-q-TOF MS/MS instrument (Bruker Daltonics Inc., Billerica, MA, USA) equipped with an Apollo II design ESI source (Bruker Daltonics Inc., Billerica, MA, USA) in positive ion mode was utilized to obtain lipid profiles. The ionization source parameters included a 4500 V capillary voltage, 800 V end plate offset, 4.0 bar nebulizer pressure, 4.0 L/min dry gas, and 250 °C dry heater. All spectra were mobility and m/z internally calibrated using a tuning mix calibration standard from Agilent Technologies (Santa Clara, CA, USA). TIMS-q-TOF MS/MS allowed for parallel accumulation serial fragmentation (PASEF) for fatty acid assignment based on MS/MS. More than 200 lipids were identified using Bruker Compass MetaboScape Version 8.0.1 and the spectral library from MetaboBASE (Bruker Daltonics Inc., Billerica, MA, USA). For assessment of ZEA, lipids were grouped into corresponding classes and quantified with statistical analysis, including a *t*-test to compare concentrations of solvent-only (0.0001% ethanol) control and treated embryos (see Figure 6).

### 5.7. Visualization of ROS in Zebrafish Embryos 

To assess the role of oxidative stress, the generation of reactive oxygen species (ROS) was visualized, specifically based on the fluorescence of intracellularly oxidized 2′,7′-dichlorofluorescein, in intact zebrafish embryos, as previously described [28,29], following 24 h exposure to 1 ppm ZEA, alongside solvent-only (0.0001% ethanol) controls. Acquisition of images was performed using an inverted laser-scanning confocal microscope (Leica DMi8/TL LED, Leica Microsystems CMS, Wetzlar, Germany) with an excitation wavelength of 485 nm and emission wavelength of 530 nm, using a Leica HC PL Apo CS2 (5×/0.15 Dry) objective and Leica Application Suite X (LAS X) software package, version 3.1.5. 

## Supplementary Materials

The following supporting information can be downloaded at: https://www.mdpi.com/article/10.3390/toxins15060397/s1, Figure S1: Proton (^1^H) HRMAS NMR spectra of 72-h post-fertilization embryos of zebrafish exposed to 1 ppm ZEA; Figure S2: Proton (^1^H) HRMAS NMR spectra of 72-h post-fertilization embryos of Olive Flounder exposed to 1 ppm ZEA; Figure S3: Proton (^1^H) HRMAS NMR spectra of 72-h post-fertilization embryos of Yellowtail Snapper exposed to 1 ppm ZEA; Table S1: Concentration of metabolites measured in embryos of Zebrafish, Olive Flounder and Yellowtail Snapper exposed to 1 ppm zearalenone. Rearing and breeding of flounder and snapper, as well as subsequent experimental procedures, were performed according to protocols approved by the University of Miami’s Institutional Animal Care and Use Committee (UM IACUC Protocol #20-138).
