# Supplementary material for: High-Resolution Magic Angle Spinning (HRMAS) NMR Identifies Oxidative Stress and Impairment of Energy Metabolism by Zearalenone in Embryonic Stages of Zebrafish (Danio rerio), Olive Flounder (Paralichthys olivaceus) and Yellowtail Snapper (Ocyurus chrysurus)
Source: Toxins (Basel). 2023 Jun 15;15(6):397. doi: 10.3390/toxins15060397 (PMC10304933; doi:10.3390/toxins15060397)
Supplement: Supplementary file 1 [file toxins-15-00397-s001.zip › toxins-2408113-supplementary.pdf]

toxins

MDPI

Supplementary Materials: High-Resolution Magic Angle Spinning (HRMAS) NMR Identifies Oxidative Stress and Impairment of Energy Metabolism by Zearalenone in Embryonic Stages of Zebrafish (*Danio rerio*), Olive Flounder (*Paralichthys olivaceus*) and Yellowtail Snapper (*Ocyurus chrysurus*)

Mark Annunziato, Narmin Bashirova, Muhamed N. H. Eeza, Ariel Lawson, Daniel Benetti, John D. Stieglitz, Jörg Matysik, A. Alia and John P. Berry

Table S1. Concentration of metabolites measured in embryos of Zebrafish (*Danio rerio*), Olive Flounder (*Paralichtyhus olivaceus*) and Yellowtail Snapper (*Ocyurus chrysurus*) exposed to 1 parts-per-million (ppm) zearalenone ("Exposed"), compared to embryos exposed to solvent vehicle, i.e., 0.0001% ethanol, only ("Control"), as measured by high-resolution magic angle spinning NMR (HRMAS NMR). Given are average concentrations (± one standard deviation), N = 6; except Flounder control, N = 3. Values indicated by "---" were not detected or able to be resolved. Concentration was measured based on reference signal of TSP.

Concentration (mM)

	Zebrafish		Flounder		Snapper	
Metabolite	Control	Exposed	Control	Exposed	Control	Exposed
**αKg**	0.093 ±0.004	0.094 ±0.014	0.008 ±0.002	0.004 ±0.003	0.018 ±0.001	0.018 ±0.001
**ADP**	0.18 ±0.006	0.441 ±0.048	0.021 ±0.002	0.075 ±0.024	0.011 ±0.001	0.018 ±0.003
**ATP**	0.309 ±0.007	0.153 ±0.022	0.091 ±0.008	0.046 ±0.011	0.037 ±0.005	0.021 ±0.004
**Ace**	1.332 ±0.023	1.33 ±0.039	---	---	0.015 ±0.001	0.015 ±0.002
**Ala**	1.826 ±0.043	1.799 ±0.030	0.078 ±0.012	0.063 ±0.011	0.02 ±0.003	0.018 ±0.002
**Ans**	0.151 ±0.003	0.149 ±0.019	---	---	---	---
**Asn**	0.806 ±0.059	0.755 ±0.085	0.128 ±0.021	0.118 ±0.018	0.02 ±0.002	0.018 ±0.003
**Asp**	0.279 ±0.009	0.288 ±0.010	0.116 ±0.032	0.116 ±0.023	0.018 ±0.001	0.018 ±0.003
**Carn**	0.12 ±0.008	0.101 ±0.002	---	---	---	---
**Carno**	0.779 ±0.036	0.646 ±0.033	0.119 ±0.01	0.047 ±0.02	---	---
**Cho**	0.356 ±0.034	0.223 ±0.009	0.082 ±0.009	0.017 ±0.005	0.006 ±0.001	0.004± <0.001
**Cit**	0.249 ±0.017	0.229 ±0.025	0.015 ±0.006	0.013 ±0.003	0.012± <0.001	0.013 ±0.004
**CRN**	0.043 ±0.003	0.037 ±0.006	0.004 ±0.002	0.005 ±0.001	0.004 ±0.001	0.001 ±0.001
**Glc**	1.018 ±0.021	1.688 ±0.138	0.213 ±0.033	0.389 ±0.036	0.029 ±0.005	0.048 ±0.004
**G1P**	0.401 ±0.011	0.29 ±0.006	0.356 ±0.041	0.127 ±0.048	0.026 ±0.003	0.008 ±0.002
**G6P**	0.832 ±0.028	1.454 ±0.033	0.216 ±0.012	0.336 ±0.02	0.009 ±0.002	0.018 ±0.004
**Glu**	1.478 ±0.040	1.528 ±0.063	0.193 ±0.02	0.199 ±0.014	0.015 ±0.004	0.033 ±0.005
**Gln**	1.391 ±0.043	1.425 ±0.051	0.658 ±0.078	0.623 ±0.056	0.005 ±0.002	0.021 ±0.008
**GSH**	0.506 ±0.009	0.229 ±0.028	0.171 ±0.017	0.056 ±0.033	0.013 ±0.001	0.006 ±0.001
**Gly**	1.268 ±0.008	1.055 ±0.115	0.197 ±0.043	0.045 ±0.014	0.031 ±0.005	0.018 ±0.002

**Lac**	2.803 ±0.009	3.213 ±0.076	0.069 ±0.009	0.14 ±0.026	0.012 ±0.001	0.016 ±0.001
**Lys**	1.283 ±0.023	1.239 ±0.083	0.216 ±0.024	0.081 ±0.024	0.005 ±0.001	0.005 ±0.001
**Mal**	0.198 ±0.006	0.174 ±0.031	0.015 ±0.006	0.009 ±0.002	0.013 ±0.003	0.014 ±0.002
**NAA**	1.005 ±0.014	1.142 ±0.138	0.158 ±0.035	0.129 ±0.026	0.013 ±0.001	0.014 ±0.001
**AcLys**	1.189 ±0.058	1.205 ±0.150	0.229 ±0.03	0.251 ±0.019	---	---
**NADH**	0.53 ±0.032	0.354 ±0.032	0.214 ±0.012	0.095 ±0.036	0.015 ±0.001	0.004 ±0.001
**PChol**	0.08 ±0.006	0.08 ±0.005	---	---	---	---
**Phe**	0.373 ±0.013	0.379 ±0.033	0.044 ±0.024	0.066 ±0.009	0.017 ±0.002	0.029 ±0.005
**Pyr**	0.95 ±0.009	0.623 ±0.012	0.164 ±0.029	0.038 ±0.015	0.02 ±0.001	0.014 ±0.003
**Succ**	0.21 ±0.005	0.238 ±0.030	0.042 ±0.016	0.038 ±0.012	0.01 ±0.003	0.012 ±0.002
**Tau**	0.712 ±0.026	0.701 ±0.035	0.072 ±0.007	0.013 ±0.004	0.009 ±0.001	0.016 ±0.006
**TMAO**	1.698 ±0.022	1.476 ±0.161	---	---	0.005± <0.001	0.003 ±0.001
**Trp**	0.276 ±0.005	0.285 ±0.027	0.164 ±0.02	0.068 ±0.033	0.011 ±0.002	0.015 ±0.001
**Tyr**	0.991 ±0.031	0.966 ±0.043	0.202 ±0.026	0.202 ±0.013	0.023 ±0.004	0.034 ±0.005
**mIns**	0.62 ±0.024	0.644 ±0.098	---	---	0.026 ±0.001	0.026 ±0.002
**GPC**	0.634 ±0.040	0.497 ±0.041	---	---	0.005 ±0.001	0.003 ±0.001

Abbreviations: Pyruvate (Pyr), lactate (Lac), glucose-6-phosphate (G6P), glucose-1-phosphate (G1P), glucose (Glc), adenosine diphosphate (ADP), adenosine triphosphate (ATP), reduced nicotinamide adenine dinucleotide (NADH), a-ketoglutarate (KG), citrate (Cit), malate (Mal), succinate (Succ), alanine (Ala), asparagine (Asn), aspartate (Asp), glutamate (Glu), glutamine (Gln), glycine (Gly), lysine (Lys), phenylalanine (Phe), tryptophan (Trp), tyrosine (Tyr), taurine (Tau), choline (Cho), glycerophosphorylcholine (GPC), O-phosphocholine (PChol), acetate (Ace), carnitine (Carn), reduced glutathione (GSH), carnosine (Carno), N-acetylaspartate (NAA), anserine (Ans), acetyllysine (AcLys), myo-inositol (mIns) and trimethylamine-N-oxide (TMAO).

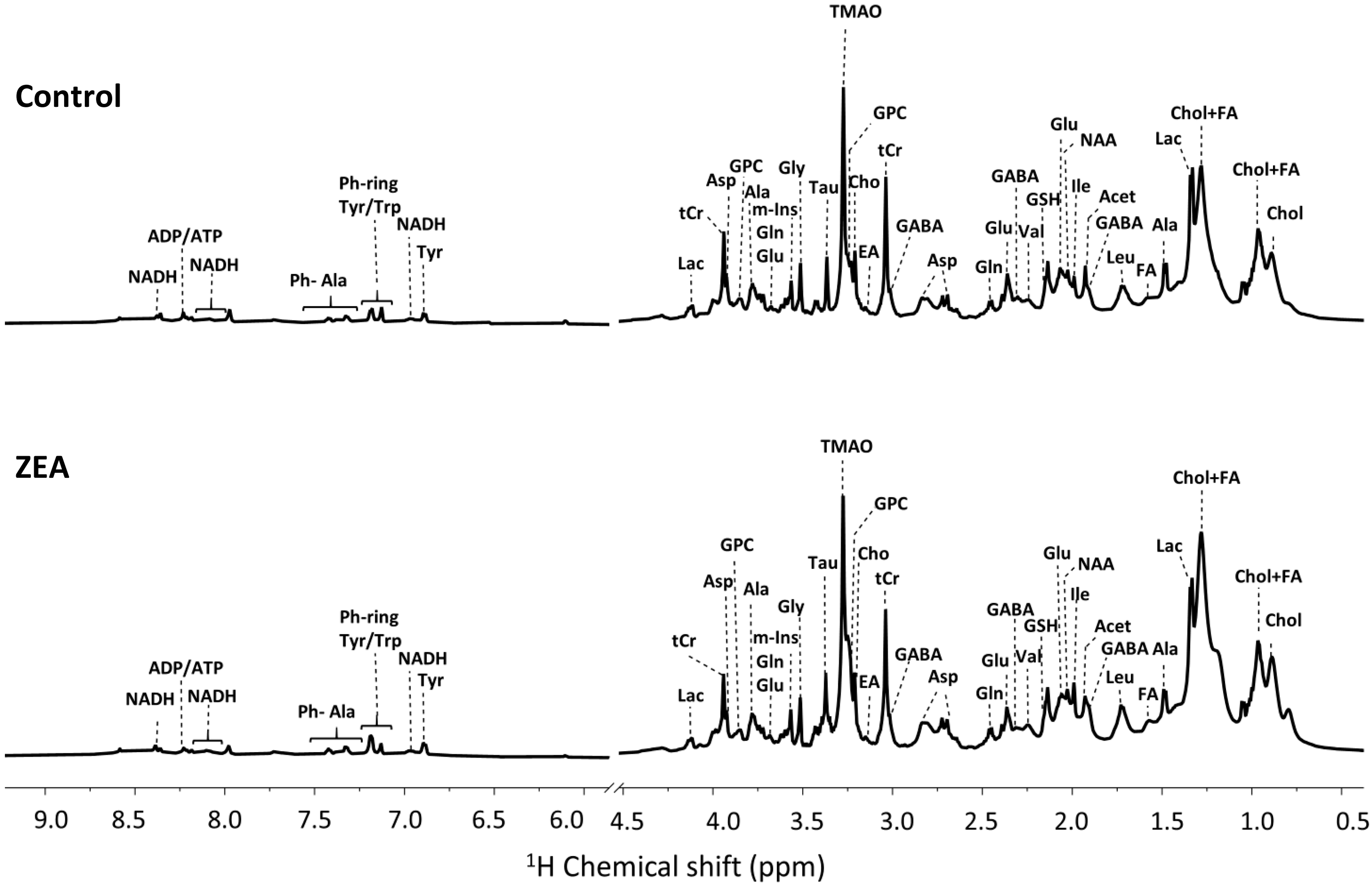

Figure S1. Proton (^{1}H) HRMAS NMR spectra of 72-hour post-fertilization (hpf) embryos of zebrafish exposed to 1 ppm zearalenone (ZEA) for 24 h, and control embryos ("Control") exposed to solvent vehicle, 0.001% ethanol, only for 24 h. Relevant signals used to identify and quantify metabolites are given. Solvent peak between 4.5 and 6 ppm removed from spectrum. For quantitation, and list of abbreviations, see Table S1.

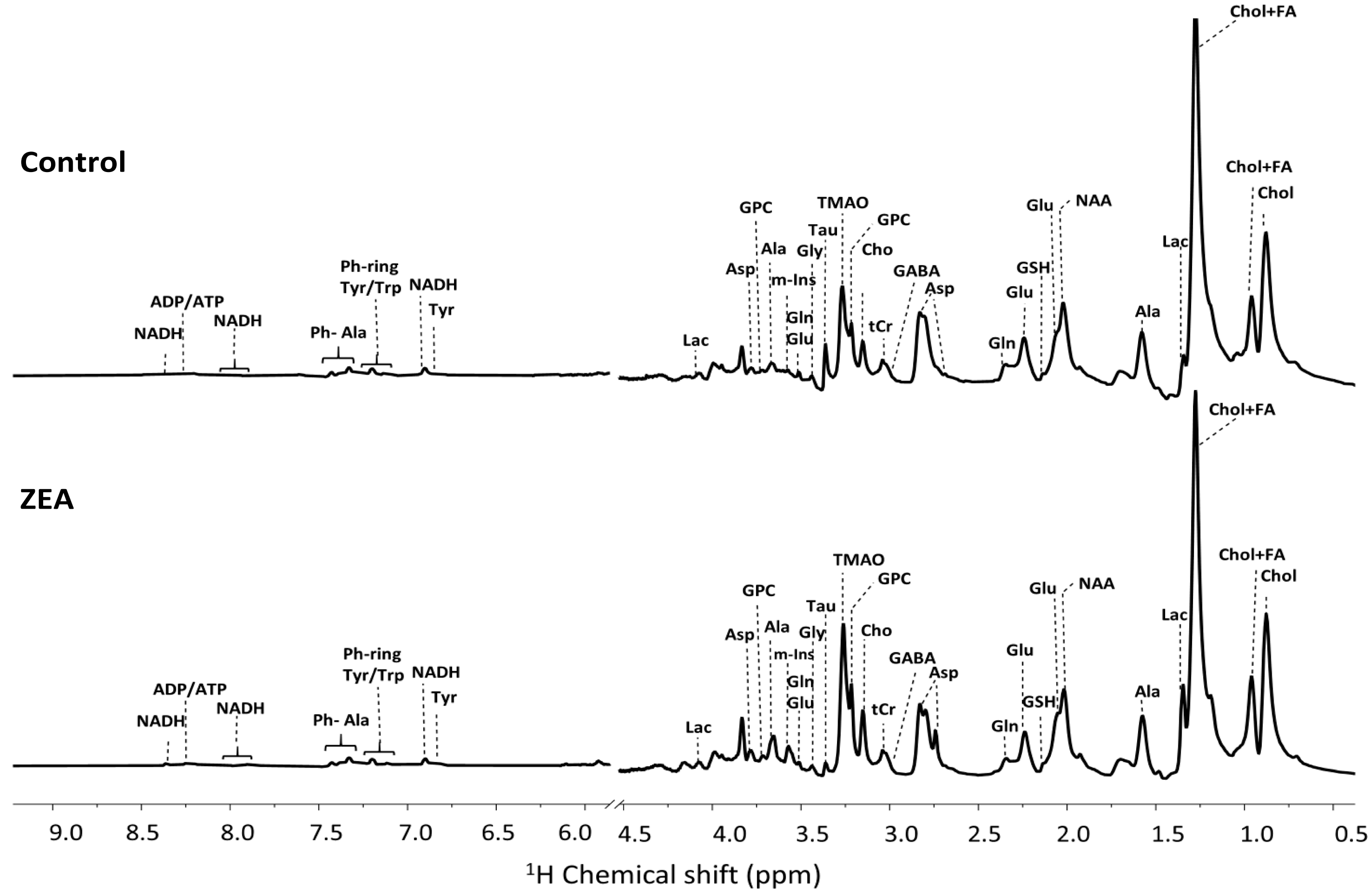

Figure S2. Proton (^{1}H) HRMAS NMR spectra of 72-hour post-fertilization (hpf) embryos of Oliver Flounder exposed to 1 ppm zearalenone (ZEA) for 24 h, and control embryos ("Control") exposed to solvent vehicle, 0.001% ethanol, only for 24 h. Relevant signals used to identify and quantify metabolites are given. Solvent peak between 4.5 and 6 ppm removed from spectrum. For quantitation, and list of abbreviations, see Table S1.

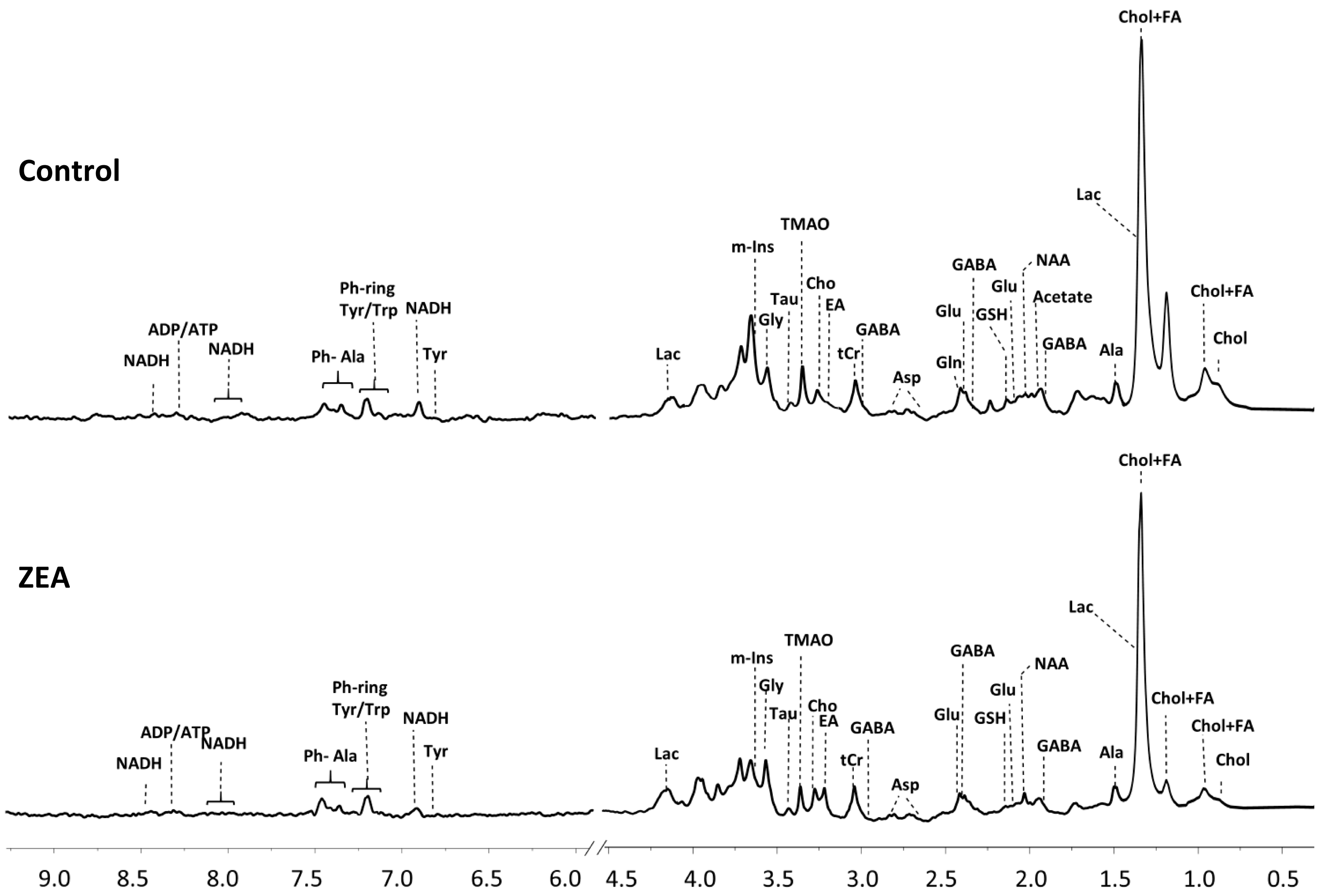

Figure S3. Proton (^{1}H) HRMAS NMR spectra of 72-hour post-fertilization (hpf) embryos of Yellowtail Snapper exposed to 1 ppm zearalenone (ZEA) for 24 h, and control embryos ("Control") exposed to solvent vehicle, 0.001% ethanol, only for 24 h. Relevant signals used to identify and quantify metabolites are given. Solvent peak between 4.5 and 6 ppm removed from spectrum. For quantitation, and list of abbreviations, see Table S1.
